# Cognitive Functions in Ataxia with Oculomotor Apraxia Type 2

**DOI:** 10.3389/fneur.2012.00125

**Published:** 2012-08-10

**Authors:** Peter Klivényi, Dezso Nemeth, Tamas Sefcsik, Karolina Janacsek, Ildiko Hoffmann, Gabor Peter Haden, Zsuzsa Londe, Laszlo Vecsei

**Affiliations:** ^1^Department of Neurology, University of SzegedSzeged, Hungary; ^2^Institute of Psychology, University of SzegedSzeged, Hungary; ^3^Department of Linguistics, University of SzegedSzeged, Hungary; ^4^Hungarian Academy of SciencesBudapest, Hungary; ^5^Department of Applied Linguistics, University of CaliforniaLos Angeles, CA, USA

**Keywords:** ataxia oculomotor apraxia 2, cognition, neuropsychology, implicit sequence learning, working memory, fluency, executive functions, speech

## Abstract

**Background:** Ataxia with oculomotor apraxia type 2 (AOA2) is characterized by cerebellar atrophy, peripheral neuropathy, oculomotor apraxia, and elevated serum alpha-fetoprotein (AFP) levels. The disease is caused by a recessive mutation in the senataxin gene. Since it is a very rare cerebellar disorder, no detailed examination of cognitive functions in AOA2 has been published to date. The aim of the present study was to investigate the neuropsychological profile of a 54-year-old patient with AOA2. **Methods:** A broad range of neuropsychological examination protocol was administered including the following domains: short-term, working- and episodic-memories, executive functions, implicit sequence learning, and the temporal parameters of speech. **Results:** The performance on the Listening Span, Letter Fluency, Serial Reaction Time Task, and pause ratio in speech was 2 or more standard deviations (SD) lower compared to controls, and 1 SD lower on Backward Digit Span, Semantic Fluency, articulation rate, and speech tempo. **Conclusion:** These findings indicate that the pathogenesis of the cerebrocerebellar circuit in AOA2 is responsible for the weaker coordination of complex cognitive functions such as working memory, executive functions, speech, and sequence learning.

## Introduction

Ataxia with oculomotor apraxia type 2 (AOA2) was first described in 1998 in a Japanese family (Watanabe et al., 1998). This autosomal recessive, slowly progressing ataxia is characterized by cerebellar symptoms, oculomotor apraxia, elevated serum alpha-fetoprotein (AFP) levels, and peripheral neuropathy. Other clinical features include involuntary movements, dystonia, tremor, and elevated serum creatine kinase (CK) levels. The typical age of onset is between 3 and 30 years of age. The available data indicate marked cerebellar atrophy, loss of Purkinje cells, and demyelinization. A mutated gene (senataxin – SETX) on chromosome 9q34 has been identified in AOA2. SETX encodes SETX protein with an unknown function (Watanabe et al., 1998; Nemeth et al., 2000; Le Ber et al., 2004). Similar autosomal, but dominantly inherited conditions are the spinocerebellar ataxias (SCAs). SCA is a heterogenous group with several different subtypes and with different gene mutations. AOA2 and SCAs share ataxia as a common feature, with additional symptoms (pyramidal tract, basal ganglia involvement, epilepsy, etc.), making them clinically indistinguishable from each other. Therefore, the diagnosis is primarily based on genetic testing (for a recent review see Matilla-Duenas et al., 2012).

In AOA2, subtle cognitive changes have been reported (Le Ber et al., 2004) based on the Mini-mental State Examination (MMSE; Folstein et al., 1975) and the Mattis Dementia Rating Scale (MDRS; Mattis, 1988), but no detailed cognitive neuropsychological analysis of AOA2 has been published to date. Le Ber et al. (2004) examined only executive functions measured by Wisconsin Card Sorting (WCST) and Verbal Fluency task, and verbal short-term memory by California Verbal Learning Task and found mild to moderate impairments in AOA2 patients. In Leggio et al.’s (2008) study, two participants had AOA2, but they were not analyzed separately, instead collapsed into a SCA group. Leggio et al. (2008) found impairments in sequence detection and production (i.e., cognitive sequencing) which are relevant in different motor, sensory, and higher-order cognitive domains, and are thought to be the primary function of cerebellum (e.g., Doyon et al., 1998). As the neuropathological background of the AOA2 disease is not fully known and the characteristic clinical features are cerebellar symptoms, cognitive changes similar to other cerebellar lesions, such as the SCA can be assumed to be present. A prominent executive dysfunction was demonstrated in SCA1, while deficits of verbal memory and attention were mild in SCA1, SCA2, and SCA3 (Burk et al., 1999; Burk et al., 2001; Le Pira et al., 2002; Burk et al., 2003). The neuropathological differences in SCA types suggest that these cognitive deficits may not only be related to the cerebellar degeneration but may reflect the involvement of the cerebellar dentate nucleus and the striatal nuclei as well (Burk et al., 2003).

The aim of our exploratory study was to investigate a broader range of neuropsychological functions such as short-term, working, and long-term episodic memory, executive functions, implicit sequence learning, and temporal parameters of speech in a patient with AOA2. Our study goes beyond previous studies in the following ways: (1) we investigate different aspects of memory compared to Le Ber et al. (2004), and (2) cognitive sequencing is also tested with implicit sequence learning and spontaneous speech analysis. Since it is a rare neurological condition we present our investigations as a case study.

## Materials and Methods

### Participants

The patient was a 54-year-old left handed male. The control group consisted of four healthy participants, matched on age, education, and gender. The intellectual ability was measured by the Raven Standard Progressive Matrices (SPM) Test (Raven et al., 2004). Both the patient and the control group were in the normal IQ range (raw scores: 58 vs. 56.25 ± 0.96, respectively), and a had maximum score (30) on the MMSE (Folstein et al., 1975). The study was approved by the local ethical committee of the University of Szeged. All participants were informed about the methods and aims of the study, and gave their written informed consent.

The patient was a Caucasian male, born after a normal pregnancy and delivery. His family history was negative for neurological diseases. Starting at 51 years of age imbalance and gait ataxia appeared, followed by the development of limb ataxia and dysarthria. His handwriting changed. At the time of the study he was able to walk independently with unilateral support.

Neurological examination revealed that both hypo- and hypermetric saccadic eye movements were present. His speech was dysarthric. His intonation indicated a foreign accent syndrome (FAS). FAS is a rare motor speech disorder resulting in articulatory distortions that are perceived by native-speakers as a foreign accent (Marien et al., 2006, 2009). Marien et al.’s found that FAS is remarkably similar to ataxic speech disturbances connected to lesions of the cerebellum. However, FAS is distinct in both its characteristics and underlying mechanism from a dysarthria, and an aphasic speech output disorder (Blumstein and Kurowski, 2006).

The neurological examination also revealed that the resting muscle tone was normal and the power was 5/5 on both sides. There was no loss of sensation, the tendon reflexes were normal, and there was no pathological reflex present. On the upper limb intention tremor and mild ataxia were detectable, while on the lower limb the ataxia was severe. Due to the severe trunk ataxia his gait was unsteady. He reported autonomic dysfunction such as orthostatic hypotension, urinary incontinence, and impotence. He was able to ambulate but needed help in his daily activities.

The magnetic resonance imaging demonstrated a pronounced cerebellar and mild cortical atrophy. The SPECT scans showed normal postsynaptic D2 receptor status and normal presynaptic dopamine transporter status. The AOA2 diagnosis was confirmed by genetic analysis in Rostock, Germany (Centogene, GmbH) based on the elevated AFP levels.

### Tasks

Four neuropsychological functions were investigated: (1) memory functions, (2) executive functions, (3) implicit sequence learning, and (4) the temporal speech parameters.

#### Memory functions

##### Phonological short-term memory

Verbal short-term memory (or “phonological loop” capacity) was measured by the Digit Span Task, and the Word Span Task. The Digit Span Task involves the presentation of spoken sequences of digits (1/s) by researcher for immediate serial recall by the participant (Isaacs and Vargha-Khadem, 1989; Racsmány et al., 2005 see for Hungarian version). Starting with three-item sequences a maximum of four lists were presented at each length. If the first three lists at a particular sequence length were correctly recalled, the list length was increased by one. The maximum number of digits recalled correctly three times provided the measure of the digit span (a simple number, e.g., 6). In the Word Span Task (Nemeth et al., 2011) the process is similar but instead of numbers simple and complex words are used. The task is to correctly recall single and morphologically complex words. There were three subtypes of the task: two lists were composed of two-syllable words and one list was composed of three-syllable words, using only common and likely familiar words. No item was repeated more than once across trials within the test. In all three lists the number of words recalled correctly constituted the score.

##### Visuo-spatial short-term memory

Corsi Block Tapping Task (Lezak, 1995) was used for measuring visuo-spatial short-term memory, which is a non-verbal memory test. In this non-verbal task, nine three-dimensional black cubes are arranged on a blackboard and the participant must tap the cubes in the same order as the experimenter. Similarly to the Digit Span and Word Span Tests, the length of the sequence increases by one after three out of four successful repetitions.

##### Working memory

Working memory refers to the ability we have to hold and manipulate information simultaneously over short periods of time (Baddeley, 1992). Working memory was measured by the Backward Digit Span, Listening Span, and Reading Span Tasks. The Backward Digit Span Task (Isaacs and Vargha-Khadem, 1989) employed the same procedure as the Digit Span Task except that participants attempted to recall the sequence of the orally presented digits in a reverse order. In the Reading Span Task (Daneman and Carpenter, 1980; Racsmány et al., 2005 see for Hungarian version) participants are required to read aloud increasingly longer sequences of sentences and recall the final word of all the sentences in the correct order. Participants’ working memory capacity was defined as the longest list length at which they were able to recall all the final words. The Listening Span Task is similar to the Reading Span Task, except that in this task the experimenter reads aloud the sentences and participants have to judge whether the sentence is semantically correct or not (Daneman and Blennerhassett, 1984; for Hungarian version see Janacsek et al., 2009), and recall the last words of the sentences.

##### Rivermead behavioral memory test

The RBMT is a well-known test to investigate long-term every-day memory, especially episodic memory (Wilson et al., 1989). We administered the following sub-tests of RBMT: remembering a name, location of a hidden item, an appointment, verbal information from a news report, objects, and faces from picture cards, a short route, and orientation questions.

#### Executive functions

##### Verbal (letter and semantic) fluency task

Verbal fluency is a commonly used neuropsychological test to investigate executive functions. This task relies on the ability to produce words spontaneously within a fixed time span (Troyer et al., 1997, 1998). There are two types of the verbal fluency tasks, i.e., letter (or “phonemic”) fluency and semantic (or “category”) fluency. For letter fluency, words must be produced according to phonemic constraints (i.e., exemplars beginning with a specified letter). For category fluency, words must be produced according to semantic constraints (i.e., exemplars which belong to a specific semantic category, such as “animal”). The letter fluency task is a powerful tool to detect frontostriatal pathology, while category fluency tasks might correspond with fronto-temporal deficits (Spreen and Strauss, 1991; Hodges and Patterson, 1995; Troyer et al., 1998). In both tasks, verbal fluency is defined as the number of words correctly generated within 60 s.

##### Wisconsin card sorting test

This task is one of the most specific tests of the executive functions (Heaton et al., 1993; Anokhin et al., 2010). Participants are required to derive a correct card sorting rule based on a trial-by-trial feedback. As the rule changes without warning, the participant needs to modify the previously learned response strategy on the basis of the feedback information. A key indicator of cognitive flexibility is the number of perseverative errors that occur when the participant persists in using the old strategy despite the negative feedback. The poor performance on categories completed and the number of perseverative errors indicate problems with frontal lobe and frontostriatal circuits.

#### Implicit sequence learning

*Serial Reaction Time (SRT) Task* (Nissen and Bullemer, 1987) was used to measure implicit sequence learning. Most studies of sequence learning highlight the role of the basal ganglia and cerebellum (Hikosaka et al., 1999; Peigneux et al., 2000; Daselaar et al., 2003; Kincses et al., 2008; Sefcsik et al., 2009; Rieckmann et al., 2010) in this process. In the SRT task a stimulus appears on one of four vertical lines placed on the bottom of the screen and participants are required to press the corresponding key (Y, C, B, M on Hungarian keyboard) as quickly and accurately as they can. Participants are told that stimuli are appearing randomly, but, in fact, the appearance of the stimuli follows a predetermined serial order. There were 11 blocks of stimuli presented with the 12-element repeated pattern in each block (a total of 60 responses/block) and the last block (random block) differed from the previous ones. There was a half minute pause between blocks.

**Figure 1:**
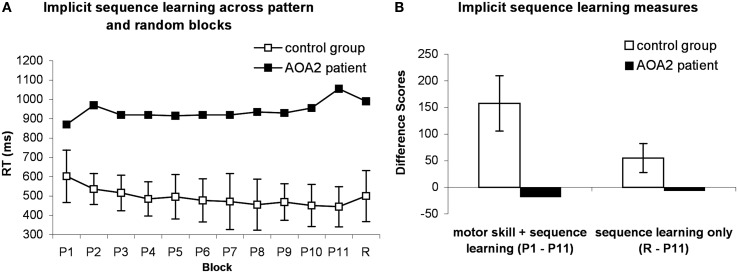
**The performance of the patient and controls on the SRT task**. **(A)** Means of median RTs are displayed for control group (open squares) and median RTs for the patient (filled squares). **(B)** Difference scores of overall skill learning (P1-P11) and sequence learning (R-P11) are displayed. Error bars indicate standard deviations, computed for control group only. P, pattern; R, random.

The reaction time (RT) is the dependent measure of primary interest in the SRT task, because the accuracy rates usually demonstrate ceiling effects (Negash et al., 2007). Gradual reduction in RTs across sequence blocks is expected to occur due to the participants’ growing expertise in learning not only the sequence but also in learning the visuomotor association between the position of the visual cue and the required motor response (Poldrack et al., 2005; Robertson, 2007). A more specific measure of learning in this task can be obtained by comparing performance on the sequence and the random blocks. Any response time advantage gained by having learned the motor response will remain during the random block, thus the difference in response times between the blocks with repeated sequences vs. random sequences will be a consequence of the participant having learned the underlying sequence patterns. When the sequence is unexpectedly substituted with a random sequence, the participant will have an inclination to respond according to the pattern learned during trials containing repeated sequences. This impulse causes RTs to increase in random trials compared to repeated sequence trials, as the participant must (implicitly) correct for this tendency (Robertson, 2007). Knopman and Nissen (1991) identified two learning measures: the RT difference between the first and the last pattern blocks (P1 and P11 in Figure 1A), which indicates both general motor skill and sequence-specific learning, and the RT difference between the last sequence block and the random block (P11 and R in Figure 1A), which assesses sequence-specific learning. These measures are commonly used in SRT research (e.g., Green et al., 1997; Westwater et al., 1998; Negash et al., 2007). Our analyses followed the formats of these studies.

#### Temporal parameters of speech

To investigate speech production, the experimenter engaged in a guided conversation with each participant. While participants were able to respond freely, each was asked the same questions. These conversations were recorded, phonetically transcribed, and an oscillogram was created. The articulation rate, the speech tempo, and the pause ratio were measured (Feldstein and Bond, 1981; Hoffmann et al., 2010). The articulation rate was defined as the total number of phonemes produced (by the participant) during the conversation divided by the total conversation time (participant’s production only) with all pauses omitted. The speech tempo is similar to the articulation rate, except that pauses are included in the total conversation time. The pause ratio is the ratio of the duration of all pauses to participant’s total conversation time, including all pauses (Sefcsik et al., 2009; Hoffmann et al., 2010).

## Results

### Memory functions

In the case of verbal short-term memory (Table 1), the patient’s performance on the simple Word Span (4.0 vs. 4.5 ± 0.58), Morphologically Complex 2-Syllable Word Span (4.0 vs. 4.5 ± 0.65), and Morphologically Complex 3-Syllable Word Span (4.0 vs. 3.87 ± 0.25) task was within 1 standard deviation (SD) of the controls’. Similarly, the performance on the Digit Span Task was comparable to controls (5.00 vs. 6.2 ± 1.3). One-sample *t*-tests for controls’ data compared to patient’s datapoint were not significant in either cases (*p*’s > 0.14).

**Table 1:** **Patient and control group performance on neuropsychological tasks**.

Tasks	Patient	Controls (*n* = 4)
Digit span	5.0	6.2 ± 1.3
Word span	4.0	4.5 ± 0.58
Morphologically complex word span (2 syllables)	4.0	4.5 ± 0.65
Morphologically complex word span (3 syllables)	4.0	3.87 ± 0.25
Corsi block tapping test	6.0	5.75 ± 0.5
Backward digit span	4*	**5.0 ± 0.82**
Reading span	4.0	4.58 ± 0.92
Listening span	3**	**3.75 ± 0.32**
RBMT (screening score)	12	12 ± 0
RBMT (profile score)	24	24 ± 0
Letter fluency (words/1 min)	8.67**	**15.75 ± 1.55**
Semantic fluency (words/1 min)	17.5*	**26.75 ± 5.38**
WCST categories	6	5.69 ± 1.12
WCST total errors	11	17 ± 14.4
WCST perseverative errors	5	7 ± 5.71
SRT – motor + sequence learning (P1-11)	−18.5**	**157.5 ± 51.88**
SRT – sequence-specific learning (P11-R)	−6.5**	**55 ± 27.58**
Articulation rate (sound/s)	11.2*	**14.1 ± 2.1**
Speech tempo (sound/s)	9.8*	**13.2 ± 3.3**
Pause/signal time ratio (%)	32**	**17 ± 3.1**

More than 1 SD differences between patient and controls in bold. (*More than 1 SD, **2 or more SD difference between patient and control performance).

The visuo-spatial short-term memory, measured by Corsi Block Tapping Task showed no difference in the patient and in the controls [6 vs. 5.75 ± 0.5; *t*(3) = −1.00, *p* = 0.39]. The RBMT screening and profile scores were exactly the same as in the control group (12 and 24).

In the case of working memory, the patient’s performance was 1 SD lower on the Backward Digit Span [4 vs. 5.00 ± 0.82; *t*(3) = 2.45, *p* = 0.09] and 2 SDs lower on the Listening Span Task [3 vs. 3.75 ± 0.32; *t*(3) = 4.55, *p* = 0.02]. The patient performed at a normal level on the Reading Span Task [4.0 vs. 4.58 ± 0.92; *t*(3) = 1.30, *p* = 0.28].

### Executive functions

On the Letter Fluency Task, the patient’s performance was 4 SDs below the mean score of the control group (8.67 vs. 15.75 ± 1.55). The number of the produced words on the Semantic Fluency Task was also lower than that of the control group (17.5 vs. 26.75 ± 6.37), but to a lesser extent (1 SD below). Both difference was significant [*t*(3) = 8.96, *p* = 0.003; *t*(3) = 3.41, *p* = 0.04, respectively].

In the case of the WCST, the patient performed at a similar level as the controls (number of categories completed: 6 vs. 5.69 ± 1.12; number of total errors: 11 vs. 17 ± 14.4; perseverative errors: 5 vs. 7 ± 5.71; *p*’s > 0.47).

### Implicit sequence learning

As is typical in the SRT task, the accuracy levels remained high throughout the task. The percentage of correct responses reached 99% in all participants during the trials. Therefore, for the purpose of better performance discrimination we focused our analyses on RTs. Median RTs for correct responses within 4 SDs were calculated separately for each block for each participant. As is shown in Figure 1A, the patient was generally 4 SD slower than the control group (937.27 vs. 491.36 ± 109.69 ms). This difference was significant [one-sample *t*-test for controls’ data compared to patient’s datapoint: *t*(3) = −11.76, *p* = 0.001]. Additionally, the patient did not show a general increase in speed compared to the controls: the performance difference between the first and the last pattern blocks was high for the control group (157.5 ± 51.88 ms) and significantly different from zero [one-sample *t*-test: *t*(3) = 6.07, *p* = 0.009] and also from the patient’s performance [−18.5 ms; one-sample *t*-test: *t*(3) = 6.78, *p* = 0.007]. The patient’s performance was 3 SDs lower compared to the controls’ score, suggesting impairment in overall skill learning (Figure 1B). Similarly, the difference between the last pattern and the random block for controls (55 ± 27.58 ms) was significantly larger than zero [one-sample *t*-test: *t*(3) = 5.37, *p* = 0.013] and also larger than the patient’s performance [−6.5 ms; one-sample *t*-test: *t*(3) = 4.49, *p* = 0.02]. These results suggest sequence-specific learning for the control group only.

### Temporal parameters of speech

We found that the articulation rate of the patient was 11.2 sounds/s, which lies just below 1 SD of the mean score of the control group [*t*(3) = 2.74, *p* = 0.07]. The speech tempo was 9.8 sounds/s, which is 1 SD slower than that of controls but not significant [*t*(3) = 2.09, *p* = 0.13]. The pause/signal time ratio was 32%, which is very high because the maximum ratio was 19% in control participants. The ratio of the patient fell below 4 SDs of the mean score of the controls producing a significant difference [*t*(3) = −9.59, *p* = 0.002].

## Discussion

To the best of our knowledge, this study is the most extensive examination of cognitive functioning in a patient with AOA2 to date. We employed a more comprehensive test of short-term and working memory system than was used in previous AOA2 and SCA studies (Burk et al., 1999; Burk et al., 2001; Le Pira et al., 2002; Burk et al., 2003; Leggio et al., 2008). In addition, we performed an analysis of temporal parameters of speech (Hoffmann et al., 2010), which has not been examined in this desease so far. Our investigation revealed cognitive deficits in executive functions (measured by Verbal Fluency), working memory (measured by Listening Span and Backward Digit Span Task), temporal parameters of speech, and implicit sequence learning.

Consistent with other investigations of patients with SCA, our patient showed decreased performance on verbal fluency tests compared to controls. Impaired working memory tasks are also consistent with earlier studies of SCA (Burk et al., 1999; Burk et al., 2001; Le Pira et al., 2002; Burk et al., 2003), while normal performance on the Digit Span, Word Span, and Corsi Block Tapping Tasks suggests that the verbal and visuo-spatial short-term memory is less affected by the cerebellar impairment in AOA2 compared to different SCA types.

The temporal analysis of speech revealed a decreased articulation rate and increased pause ratio. These results are consistent with previous studies of speech in SCA and other cerebellar disease (Ackermann and Ziegler, 1991; Ackermann et al., 1992; Ackermann et al., 1999; Ackermann, 2008). In addition, the results of speech analysis are in line with the FAS of our patient. Interestingly, our AOA2 patient showed a different pattern in temporal parameters of speech compared to the performance of a patient with a focal basal ganglia lesion (Sefcsik et al., 2009). Sefcsik et al. (2009) used the same method as the present study to analyze temporal parameters of speech and found increased pause ratio but intact articulation rate in a patient with focal left putamen lesion. Based on these results, we could propose that the increased pause ratio, in combination with impaired letter fluency, points to the involvement of the cerebellum during lexical access. In both the letter fluency task and in spontaneous speech, words must be selected from the mental lexicon (Hickok and Poeppel, 2007). However, since AOA2 is characterized by motor impairment more than patients with putamen lesion, more investigations are needed to clarify these results.

Impaired performance on the sequence learning task in our AOA2 patient is in line with previous studies that found cerebellar involvement in sequence learning (Pascual-Leone et al., 1993; Doyon et al., 1997; Gomez-Beldarrain et al., 1998). Motor functions – mostly movements – rely on serial ordering and many cognitive functions (e.g., working memory, language production) do so as well. The patient’s performance on these domains support the significant role of cerebellum in cognitive sequencing (Leggio et al., 2008) and fit the classic theories of the relationship between cerebellum and cognition (Desmond and Fiez, 1998; Schmahmann, 1998; Schmahmann and Sherman, 1998).

In the sequence learning task we found neither general speed-up (index: P1-P11) nor sequence-specific learning (index: R-P11). These results raise the question whether the patient has real sequence-specific learning deficit or only general performance deficit. Using the same task Sefcsik et al. (2009) found general skill learning but no sequence-specific learning in a patient with focal putamen lesion. Based on these results, we can suggest that the basal ganglia might be responsible for the learning of the sequence structure, while the cerebellum might be responsible for the performance in coordinating the motor responses. This claim is in line with a neuroimaging study of Seidler et al. (2002) who found cerebellar activation during the expression of sequence knowledge, but not during the learning. However, further investigations are still needed to clarify the contribution of the cerebellum to the sequence learning and performance in AOA2 to decide whether there is a real learning deficit or only a deficit of the expression of the sequence knowledge.

While the cerebellum has long been associated with motor skills only, the results of our case study also support the fact that it also has a significant role in cognitive functions (Desmond and Fiez, 1998; Schmahmann, 1998; Schmahmann and Sherman, 1998), which are primarily connected to the frontostriatal circuits as well. As there is evidence that the frontostriatal areas are tightly linked to the cerebellum via the pontine structures (Allen and Tsukahara, 1974; Schmahmann and Pandya, 1995; Schmahmann, 1997), these findings can also indicate that the cerebrocerebellar circuit can be interrupted at the level of pontine (Burk et al., 2003). However, taking the limitations of the case studies into account, further investigation with a larger AOA2 population is needed to confirm our conclusions.

The primary contribution of our study is that it examined a rare cerebellar disease, AOA2, complementing previous investigations of spinocerebellar atrophies. The differences and similarities in the patterns of cognitive neuropsychological profiles among the different cerebellar disorders can help us better understand the role of the cerebellum in cognition, and better describe the cognitive profiles of the subtypes of cerebellar disorders which might help us developing new diagnostic and screening protocols in clinical practice.

## Conflict of Interest Statement

The authors declare that the research was conducted in the absence of any commercial or financial relationships that could be construed as a potential conflict of interest.
